# A New Mutation of the *Atoh1* Gene in Mice with Normal Life Span Allows Analysis of Inner Ear and Cerebellar Phenotype in Aging

**DOI:** 10.1371/journal.pone.0079791

**Published:** 2013-11-12

**Authors:** Kianoush Sheykholeslami, Vikrum Thimmappa, Casey Nava, Xiaohui Bai, Heping Yu, Tihua Zheng, Zhaoqiang Zhang, Sheng Li Li, Shuqing Liu, Qing Yin Zheng

**Affiliations:** 1 Department of Otolaryngology-HNS, Case Western Reserve University, Cleveland, Ohio, United States of America; 2 The Transformative Otology and Neuroscience Center, Binzhou Medical University, Yantai, Shandong, People's Republic of China; 3 The Jackson Laboratory, Bar Harbor, Maine, United States of America; 4 Department of Biochemistry, Dalian Medical University, Dalian, People's Republic of China; University of Washington, Institute for Stem Cells and Regenerative Medicine, United States of America

## Abstract

*Atoh1* is a transcription factor that regulates neural development in multiple tissues and is conserved among species. Prior mouse models of *Atoh1*, though effective and important in the evolution of our understanding of the gene, have been limited by perinatal lethality. Here we describe a novel point mutation of *Atoh1* (designated *Atoh1^trhl^*) underlying a phenotype of trembling gait and hearing loss. Histology revealed inner ear hair cell loss and cerebellar atrophy. Auditory Brainstem Response (ABR) and Distortion Product Otoacoustic Emission (DPOAE) showed functional abnormalities in the ear. Normal lifespan and fecundity of *Atoh1^trhl^*mice provide a complementary model to facilitate elucidation of ATOH1 function in hearing,central nervous system and cancer biology.

## Introduction

*Atoh1* (*Math1*), a basic helix-loop-helix transcription factor homolog of the *Drosophilaatonal* gene is involved in a wide range of developmental changes in mice and humans.A critical role in neurogenesis has been shown for *Atoh1* in proper hindbrain development [1]. Unconscious proprioception is dysfunctional in mice lacking *Atoh1*, with cerebella missing the external granule layer and bereft of the granule cells essential for proper functioning [2]. *Atoh1* has also been implicated in the creation and interplay of complex neural circuits that link hearing, proprioception and arousal with tasks like respiration, likely through a pathway of glutamatergic neurons [1]. Secretory cells in the gut, such as goblet, enteroendocrine and paneth cells, arise from a secretory cell lineage through a*Atoh1*-dependent pathway [3]. *Atoh1* behavesas anoncogene in medullablastomas[4] but acts as a tumor suppressorgene in adenomatous polyposiscarcinoma[5]. Apparently, the *Atoh1* gene plays many significant roles in the development of several organs, systems and diseases.

Previous studies regarding the function of the *Atoh1* gene are mostly on mutants generated from a gene-driven approach (manipulating known sequences through gene-gene targeting). It is shown that *Atoh1* is a positive regulator of hair-cell differentiation during cochlear development[6], [7].*Atoh1*-homozygous-null mice lack hair cellsin the vestibular organs and cochleae and was the first model demonstrating the phenotype associated with *Atoh1* loss of function [7].Adenovirus-mediated overexpression of *Atoh1* has been reported to produce a large number of hair cells in cultured adult utricular maculae from rats[8] and in cochleaefrom mature guinea pigs[9]. Earlier studies regarding *Atoh1* mutant mice have been limited to the prenatal stage because the targeted null mutants die shortly after birth due to respiratory failure [2]. A few studies circumvented lethality in *Atoh1* deficiency using a conditional knockout strategy [10], [11], but the authors of the Pan paper reported possible leakiness in expression resulting from incomplete recombination and a lifespan of a month or less. The conditional knockout strategy has been invaluable in understanding in vivo function of *Atoh1* and has played a significant role in helping the scientific community elucidate the primary role of *Atoh1* in development. However, some disadvantages do exist with this approach, such as the efficiency of gene deletion varying depending on the gene locus position and the Cre activity. It is also time consuming and expensive, requiring two transgenic lines (Flox and Cre mice), to generate conditional knockout mouse models [12].

In contrast to a gene-driven approach, a phenotype-driven approach to study gene function is an alternate method with a different set of advantages and drawbacks. This tactic overcomes the lethality early in life and is a similar way in which human mutation is generated. The phenotype-driven approach includes spontaneous and induced mutations such as those that are chemically induced. Here, we report anethylmethanesulfonate (EMS) induced hypomorphicmutant *Atoh1^trhl^*mouse model with a normal life span and cerebellar atrophy accompanied by a failure to form a morphologically and functionally normal inner ear. This mouse mutant could be useful model to explore human disease associated with the *Atoh1* gene due to a natural development process and having postnatal vitality. It is important to understand the expression and regulation of *Atoh1*, not just for the generation of hair cells in the inner ear, but also for the possible contribution of this gene to diseases such as medulloblastoma in which the *Atoh1* gene is thought to be improperly activated [11]. We can take advantage of this new *AtohI^trhl^* mutant mouse model to investigate many aspects of physiology, molecular biology, proteomics and the development of a variety of hearing and balance pathologies.

## Materials and Methods

### Mice

The mutant strain was first established at The Jackson Laboratory (JAX, Bar Harbor, Maine) from a mutagens program as previously described [13]. In brief, culture media contains embryonic stem (ES)cells of 129/Sv ×C57BL/6Jwere treated with the point mutagen ethylmethanesulfonate (EMS). Then the treated ES cells were injected into C57BL/6J blasto-cysts by standard methods. Chimeric mice generated from EMS-treated ES cells were mated with C57BL/6J partners to recover the recessive mutations. The trhl phenotype was first identified from the third generation mice with a ‘click box’, where mice showed a reduced Preyer reflex (pinna twitch) and then were confirmed by ABR testto have defective hearing. Progeny of later generations were noticed to have leg tremors. The *Atoh1^trhl/trhl^*mutation was moved to the C57BL/6J mouse background by backcrossing 10 generations.

Later, this mouse strain was imported to Case Western Reserve University. Mice were housed in ventilated racks at 21°C in a 12 h light–dark cycle with food and water given *ad libitum*. The mice used as control groups were either C57BL/6J wild type or *Atoh1^trhl/+^*, depending on the stage of the experiment, and were all managed in the same fashion as the *Atoh1^trhl/trhl^*mice. All animal protocolswere approved by the Institutional Animal Care and Use Committee of Case Western Reserve University (R01DC007392).

### Genotyping Assay

A DNA pooling method was used as previously described [14]. Genomic DNA was prepared from tail tips of *Atoh1^trhl/trhl^*,*Atoh1^trhl/+^*and C57BL/6J (B6)wild-type mice. Briefly, 2-mm tail tips were each digested with 0.3 ml of 50 mMNaOH in a 0.5 ml Eppendorf tube at 95°C for 10 min. A total of 26 µl of 1 M Tris-HCl was then added to each tube. The mixtures were centrifuged at 12,000 x g for 5 min and the DNA concentration in supernatants was measured using a BioPhotometer (Eppendorf AG, Hamburg, Germany). Genomic DNA screening to identify the alteration in the *Atoh1* gene was performed using PCR with primers designed to detect the *Sau*96 I site present in wild-type and absent in mutant mice: (Sau494 5′-GAAGGTGATGGTGGTCATTTTT-3′and Sau756 5′-ACAGGTGAATGGGGTACAGAAG-3′). Also,thesingle exon of the *Atoh1* gene was sequenced for detailed comparison between mutants and controls using Sequencher 4.0. (http://genecodes.com; Gene Codes Corporation, Ann Arbor, MI, USA). To confirm the mutation, reverse transcription PCR was run with initial RNA from *Atoh1* mutant and wild-type control mice. Mice around 2 months of age were sacrificed and RNA (DNA-free) was collected from the inner ears and temporal bones. cDNA was synthesized using the SuperScriptTM First-Strand Synthesis System (Invitrogen). The same gene-specific primers for the single exon of *Atoh1* were used in RT-PCR as described above for genotyping. Ten microliters of each PCR product were analyzed by agarose gel electrophoresis after exposure to the restriction enzyme *Sau*96 I.

#### cDNA preparation, RT-PCR, and quantitative RT-PCR

*Atoh1^trhl/trh1^*, *Atoh1^trhl/+^*and B6 wild-type mice were sacrificed under Avertin anesthesia conditions at 1 day or 3 days of age. The inner ears (50 mg) were quickly isolated for total RNA and cDNA preparation as described in the previously [4], [15]. One µg of total RNA from each sample was used as template for cDNA synthesis. The 20 µl reaction mixture contained 50 mMKCl, 10 mMTris-HCl, pH 9.0 (at 25°C), 0.01% Triton X-100, 2 mM MgCl_2_, 250 nM of each primer (forward and reverse), 200 µM dNTP, 1 µl of cDNA and 0.5 U of *Taq* DNA polymerase (New England Biolabs, Inc., Ipswich, MA, USA). Quantitative-RT-PCR using SYBR green PCR mix (Applied Biosystems) in an Applied Biosystems 7300 Real-Time PCR system) was performed using the following primers: GAPDH (5′-AAC TTT GGC ATT GTG GAA GG-3′ and 5′-GTC TTC TGG GTG GCA GTG AT-3′), Atoh1 (5′-GTA AGG AGA AGC GGC TGT G-3′ and 5′-AGC CAA GCT CGT CCA CTA-3′), Rab15 (5′-GGC TTG GGC TGT GTC ATT G-3′ and 5′-GGC AGA CAG GCC AGG AAA-3′), Selm (5′-TCG TGC TGT TAA GCC GAA ATT-3′ and 5′-CCG GGT CAT TTG GCT GAG T-3′), and Barhl1 (5′-GAG CGG CAG AAA TAC CTG AG-3′ and 5′-GGT CCA GAT TGG AAA CCA GA-3′).

### Generation of Cytocochleograms

Cytocochleograms were obtained bythe following method described previously[15]. Briefly, the organ of Corti was carefully microdissected out and mounted in glycerin on glass slides. The surface preparations were stained with Ehrlich's hematoxylin solution and examined under a light microscope.Hair cells were counted as present if the cell body intact. Inner and outer hair cell counts were made over 0.12-0.24-mm intervals of the organ of Corti, beginning at the apex and continuing toward the base. Individual cochleograms were constructed to show the percentage of hair cells missing as a function of distance from the apex. Composite cochleograms were generated by calculating the means at each distance point for each genotype, averaging (n = 5) mice of each genotype.

### Histology and SEM

Mice were subjected to an overdose of carbon dioxide, decapitated, and the inner ears quickly removed. The tympanic bullae were opened and a small hole was made in the round window of each exposed cochleae. 4% paraformaldehyde fixative was gently perfused through the round window, followed by immersion of cochleae in the same fixative. The cochleae were decalcified when needed for up to 6 h with Cal-Ex solution. The organ of Corti was carefully isolated by microdissection and mounted in glycerin on glass slides. The surface preparations were stained with Ehrlich's hematoxylin solution. Over 0.12–0.24 mm intervals under light microscopy (Leica DFC500, Leica Microsystems, Wetzlar, Germany) hair cells were counted as present if the cell body and cuticular plate were intact. This procedure was performed to generate the cytocochleograms both to determine where hair cells were missing along the length of the cochlea and also to examine hair cell loss over a time course.

For the silver nitrate staining, mice from each group were sacrificed by CO_2_. Organs of the cochlea and vestibular system were dissected, and the round window and oval window were pierced. Another small hole was drilled on the apical surface of the cochlea. 1.0% silver nitrate solution was gently perfused through the cochlea for 1 minute and the procedure was repeated three times. Specimens were fixed in 10% formalin for 2 h and exposed to sunlight for approximately 1 h to finish all procedures of this staining. Stained tissue was then examined under the microscope for hair bundle orientation.

Mice cerebella were dissected and embedded in paraffin wax after following the appropriate dehydration and embedding protocol. Tissue was then sectioned and stained with hematoxylin and counterstained with eosin after deparaffinizing and rehydrating sections. Tissue was also stained with cresyl violet to better view neurons [16]. Sections were then analyzed and photographed under the microscope at increasing magnifications.

For the SEM images,inner ears from *Atoh1^trhl/trhl^* and wild-type mice weredissected after transcardial perfusion with 4% paraformaldehyde (PFA) and then immersed in 2.5% glutaraldehyde in 0.1 M phosphate buffered saline (PBS, pH = 7.2) for 4 hours at 4°C. Dissection was performed to expose the middle ear cavities and inner ear basilar membrane. After post-fixation in 1% OsO4 in 0.1 M PBS (pH = 7.2, 1 hour), samples were washed in 0.1 M PBS (pH = 7.2), dehydrated in increasing concentrations of ethanol, dried in a BAL-TEC CPD 030 critical point dryer (BAL-TEC GmbH, Witten, Germany) according to manufacturer's instructions, and analyzed on a Hitachi S-4500 scanning electron microscope (Hitachi, Tokyo, Japan) at 5 kV.

### Evaluation of auditory function in mice

The ABR methodology has been previously described and includes criteria for evaluating hearing loss in response to various stimuli [17]. ABR thresholds were obtained for each stimulus (clicks, and 8, 16, and 32 kHz tone-bursts) by reducing the sound pressure level (SPL) at 10 dB steps and finally at 5 dB steps up and down to identify the lowest level at which an ABR pattern could be recognized. ABR was measured at various intervals for *Atoh1* mutant and control mice (at ages ≥14 days) up until 8 months of age (n = 5 per group).

To test the function of outer hair cells of different mice at different time points, we used DPOAE measurement, which was conducted for pure tones from 2 to 36 kHz. Frequencies were acquired with an F2-F1 ratio of 1∶22. Five stimulation levels ranging from 65 to 25 dB SPL in 10 dB steps were used to assay DPOAE at multiple ages (n = 5 per group).

## Results and Discussion

### Gross phenotype, genetic mapping and identification of the Atoh1^trhl^ mutation define the first viable normal-life-span mutant without conditional gene targeting

The *trhl*model is the result of anEMS induced hypomorphic allele of the *Atoh1* geneon the C57BL/6J (B6) mouse strain[13]. The phenotype of these hypomorphic mutant mice includes a trembling gait and hearing loss (symbol: *trhl*). Homozygous mutant mice exhibit a shaky gait at weaning age and a progressive hearing loss starting at 3 weeks of age (as we tested) or earlier and culminating in near-total deafness by 8 months, with a lifespan that is comparable to wild-type mice. This lifespan is in comparison to two previously described targeted mutations of *Atoh1*which affected inner-ear hair cells and the cerebellum as well as the arousal system, with mice homozygous for the null mutations dying of respiratory failure shortly after birth[2], [7].

To genetically map *trhl*, we generated 230 F2 progeny mice from a cross of B6-*trhl* x CAST/Ei. We used a pooled DNA strategy[1], [14], [18]to localize the mutation to chromosome 6. We then refined the map position of the mutation to 29 cM from the centromere and narrowed the candidate gene interval to a 6 cM region between *D6Mit364* and *D6Mit71* where there are three genes that related to cerebellum like ataxia. These three genes are glutamate receptor ionotropic delta 2 (*Grid2*), catenin (cadherin associated protein) alpha 2 (*Ctnna2*) and Atoh1. We tested thethree genes as a candidate gene for the*trhl*mutation by sequence comparison.

**Figure 1:**
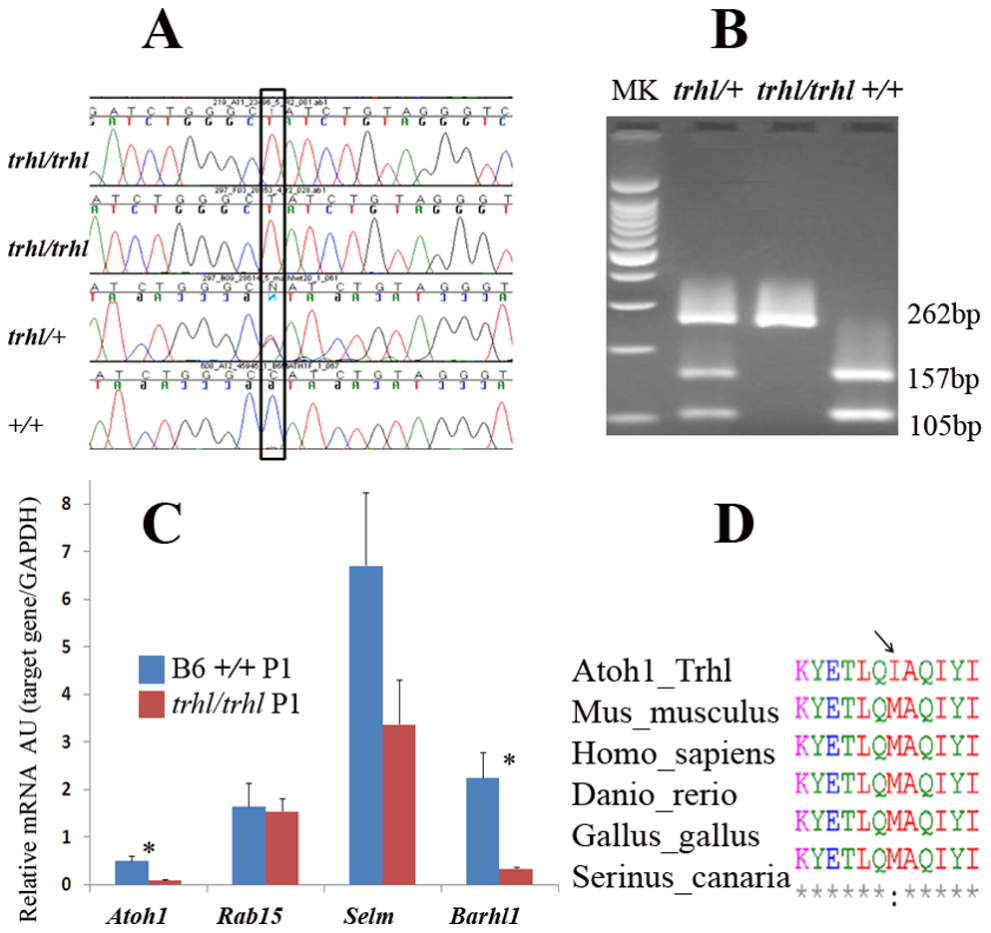
Identifying the mutation in *Atoh1^trhl/trhl^* mice. A)Sequencing comparison over the candidate gene interval reveals a G-A transition at nucleotide 600 in the 6 cM region of chromosome 6 that includes the *Atoh1* gene. The black box indicates the mutated nucleotide. **B**) The *Sau*96 I restriction site (GGNCC; from bp 596 – 600) was altered in the mutant, facilitating genotyping. Lane MK, 100 bp ladder. PCR of a 262 bp fragment in the mutation-containing region of the *Atoh1* gene, followed by *Sau*96 I digestion of the PCR product, revealed digestion of product into two smaller bands of 157 bp and 105 bp in the wild-type mice (+/+) and one intact 262 bp fragment in the homozygous mutant mice (*trhl/trhl*) due to the absence of the restriction site. The heterozygous mutant (*trhl*/*+*) displayed three bands with one mutated allele lacking the restriction site and one wild-type allele with the restriction site present. C) Gene expression levels were measured by quantitative RT-PCR. All samples were run in triplicate and normalized to the GAPDH relative expression level. The 2^−ΔΔCT^ method was used for statistical analysis. AU, arbitrary units. mRNA levels were significant lower for mutant (*trhl/trhl*) mice than wildtype at P1 for Ahoh1 and Barhl1 genes at P21 (*P<0.05). Error bars were generated by calculating the SEM, (n = 3 mice, 6 ears, at postnatal 1 day). **D**) Analysis of the conservation of methionine (M) across species and the change to isoleucine (I) in *Atoh1* mutants. M to I substitution is shown by the arrow at the 200th amino acid residue in the helix-loop-helix (HLH) DNA-binding domain of the ATOH1protein in the *trhl*mutant mouse. M in the ATOH1 protein is conserved at position 200 across species.

We first found no differences for all exons of *Grid2* and *Ctnna2*as well as 500 bp of the upstream promoter regions for both genes. Thenwe identified a G-Atransition mutation at the 600th base pair (bp) position of the *Atoh1* gene (Fig. 1A) in DNA from mutant mice.The mutation resulted in an amino acid substitution ofmethionine (M) to isoleucine (I) at the 200th amino acid residue in the helix-loop-helix (HLH) DNA binding domain, which is highly conserved between species (Fig. 1D). A*Sau*96 I restriction site was lost secondary to the mutation; accordingly, we developed a PCR assay to distinguish *trhl/+* (H), *trhl/trhl*(M)and *+/+* (W) genotypes. Primers Sau494 and Sau756 were used for PCR identification of wild-type and mutant genotypes and *Sau*96 I-digested products were run on an agarose gel (Fig. 1B). Two products were seen in the +/+ wild-type samples, which had the restriction site present, while only a single band was produced in the mutant *trhl/trhl*samples. The heterozygous mouse yields three PCR fragments, having one allele of each type. The *trhl*mutation might cause slight changes in final protein configuration and/or 3D structure of the protein with the mutation from methionine to isoleucine, whichmay allow partial development of hair cells and the central nervous system to produce a distinctive phenotype without decreasing the viability of the animal.

### mRNA expression of several genes specific to Atoh1 targets was down-regulated in the inner ear tissue from Atoh1^trhl^mice

To determine theexpression of the genes known to be direct Atoh1 targets, we examined the mRNA levels of Atoh1, Rab15, Selm and Barhl1 in inner ear tissue from thehomozygous mutant mice (*trhl/trhl* P1) (n = 3 mice, 6 ears, at 1 day) compared tothe wild-type mice (B6 +/+ P1)(n = 3 mice, 6 ears, at 1 day). The mRNA of Atoh1, and Barhl1 were significantly down-regulated in thehomozygous mutant mice compared to wild-type mice at 1 day (shown in Fig. 1C). However, there was no significant difference in mRNA levels of Rab15 in inner ear tissue between mutant mice and wild-type mice. It is widely acceptedthat *Barhl1* is one direct target of Atoh1 in the developing neural tube or cerebellum[5], [12], [19], which is sufficient for the initial generation of those hair cells even in the absence of *Barhl1*expression. The expression of Selm was down-regulated in *trhl/trhl* samples, though not significant, which were in accordance with previously report[13], indicating a G-Atransition mutation affects the regulating target genes of Atoh1.

### Histologic and scanning electron microscope examinationof hair cells

**Figure 2:**
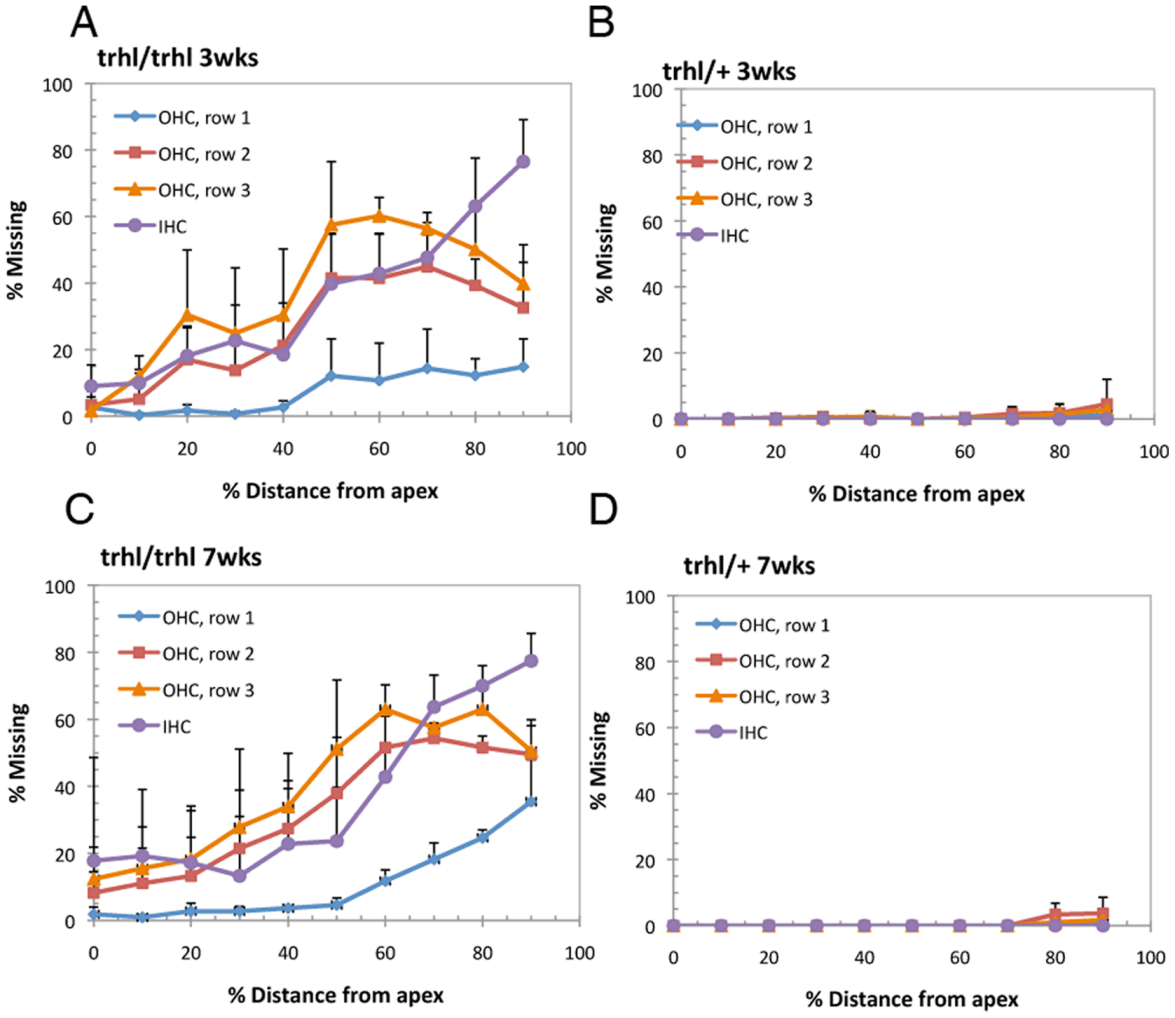
Cytochochleograms show hair cell loss in mutants. Cytocochleograms indicate percentage of outer (OHC) and inner (IHC) hair-cell loss as a function of percent distance from the cochlear apex, where 0% distance describes the most apical region of the cochlea and 100% distance describes the most basal region of the cochlea, excluding the cochlear hook. Hair-cell loss is shown for three-week-old *trhl* homozygotes (n = 5) (**A**) and heterozygotes (n = 5) (**B**) and for seven-week-old *trhl* homozygotes (n = 5) (**C**) and heterozygotes (n = 5) (**D**). Data points represent the mean percentage, with error bars indicating standard deviation. There is clear evidence of hair-cell loss to a much greater degree in the homozygous mutants as compared to the heterozygous controls.

**Figure 3:**
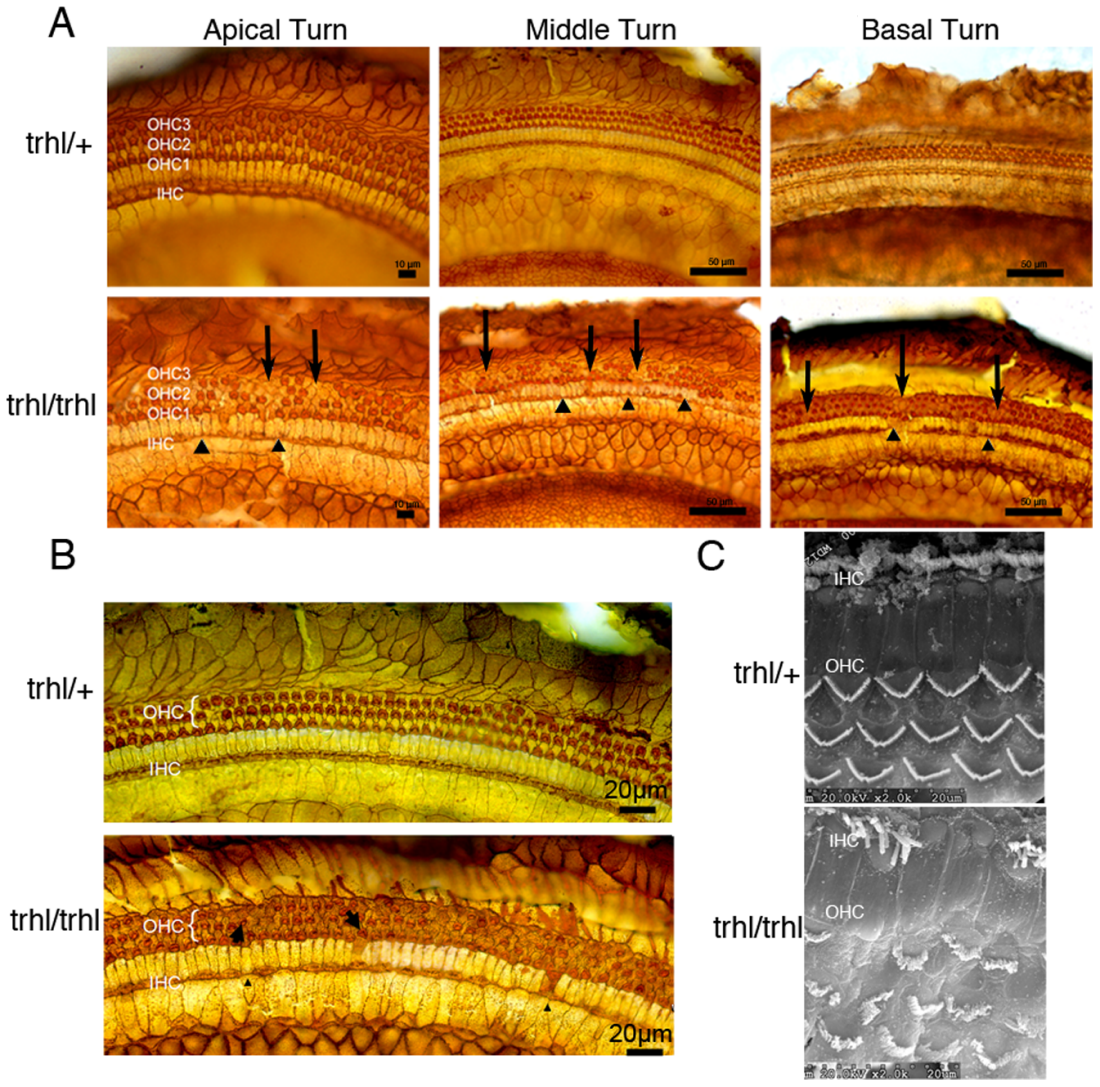
Stained cochlear sections and SEM reveal hair cell loss and altered morphology. Representative cochlear sections from*Atoh1^trhl/trhl^*and control *Atoh1^trhl/+^* mice were stained with AgNO_3_to examineOHC and IHC morphology.(**A**) At P21, sections were compared from all three turns of the cochleae in heterozygous (n = 3, representative view of *Atoh1^trhl/+^*, upper panels) and homozygous (n = 3, representative view of *Atoh1^trhl/trhl^*, lower panels) mutant mice. Arrows indicate sites of OHC absence; arrowhead/triangles indicate sites of IHC absence. (**B**) At P21, representative comparative high magnification views of sections from mutant mice and heterozygous controls are shown from the mid-turns of the cochleae, which showed the greatest degree of hair-cell loss. Arrows indicate orientation of the stereociliary bundles. Thedisorientation is clear in the *Atoh1^trhl/trhl^*mice with arrows aligned much less uniformly than in the control. (**C**) Cochleae were analyzed under the scanning electron microscopeto reveal a more detailed view of stereociliary bundle orientation. The hair-cell bundle appears as a light-colored, V-shaped (normal morphology, seen in the top panel) band within each hair cell. The lower panel shows the disorientation of bundles and missing hair cells in the homozygous mutant mice in contrast to the consistent structure of bundles in heterozygous mutant mice.

Surface preparations from mouse cochleae were stained with Ehrlich's hematoxylin solution and examined under a light microscope.Individual cytocochleograms were constructed for each mouse to show the percentage of hair cells missing as a function of distance from the apex, relative to age-matched heterozygous mice. The study revealed that in homozygous *Atoh1^trhl^*mice, outer hair cells (OHC) and inner hair cells (IHC) exhibited losses throughout the cochleae that were already present at the initial 3weeks time point, and exhibitedsimilar, but progressive, losses at 7 weeks of age as compared to heterozygote controls, shown in the composite figure using the means (Fig. 2).Hair cell loss was greatest at the mid-basal (greater than 40% distance from apex) turn of the cochleae in both three-week-old (Fig. 2A) and seven-week-old homozygous mutants (Fig. 2C). Cochlear surface preparations stained with silver nitrate (AgNO_3_) were used to examine loss of stereociliary bundles in OHC and IHC.Figure 3A shows a representative cochleae stained with silver nitrate to examine orientation and structure along the full length of the organ, with 3B showing a magnified view. In 3A, the arrows and arrowheads indicate sites of missing cells, while the arrows in 3B show direction of orientation of hair cells. There are more cells missing in the *trhl*mutant than in controls and the hair cells present are oriented haphazardly. Hair cells in the organ of Corti were viewed under SEM (Fig. 3C) to examine structure more closely. SEM views show the classic disorientation of hair bundles and missing cells in homozygous mutants as compared to the wild-type controls, confirming the inner ear morphological dysfunction of the model.

### Structural alterations in cerebella of Atoh1^trhl^mice

**Figure 4:**
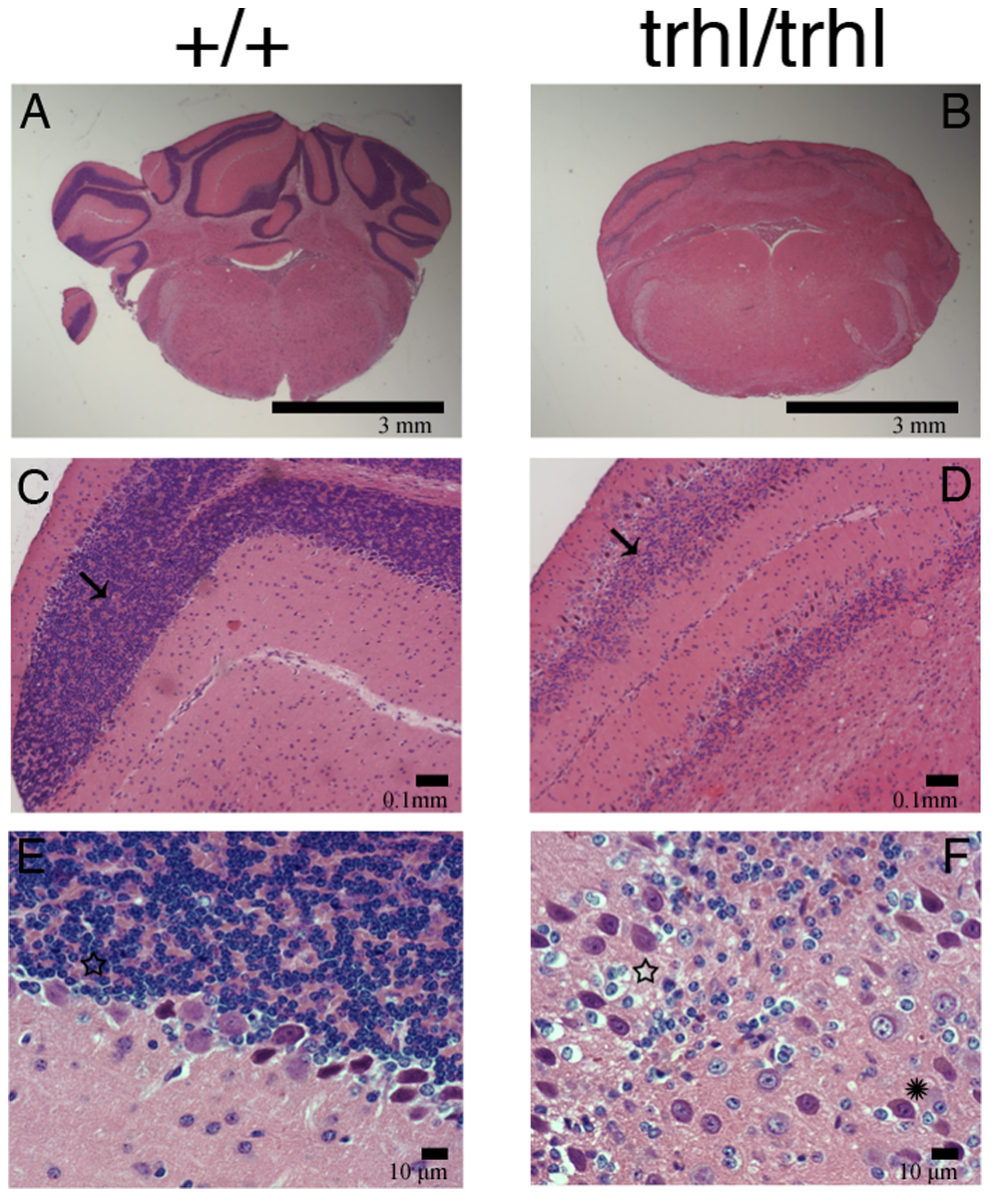
*Atoh1^trhl/trhl^*mutant compared to wild-typecontrol cerebellum morphology. Cerebellar abnormalities in H&E-stainedand cresyl violet-counterstained sections through the cerebellum of a representative wild-type control (left panels) and a *Atoh1^trhl/trhl^*(right panels) mouse shown under increasing magnification from 5X to 63X (n = 3 per group).Cerebella from each genotype were examined. The cerebellum in wild type is well developed and foliated(**A**), but it is smooth(**B**) in *Atoh1^trhl/trhl^*mice.The external granule layer (EGL)is indicated by the arrow in both middle panels. In (**D**) the EGL of the*Atoh1^trhl/trhl^*mice is significantly underdeveloped as compared to (**C**).Under high magnification, the Purkinje cell layer (PCL), indicated by the star in both (**E**) and (**F**), appears disorganized in the mutant, with some Purkinje cells (asterisk in **F**) located deep in the cerebellum,where they are not normally found.

The structure and histology of the cerebella of homozygous *Atoh1^trhl^* mice were abnormal compared to normal littermates. *Atoh1^trhl/trhl^* mice lacked robust external granular cell layers (EGL), as shown by H&E staining and cresyl violet staining. The homozygous mutant cerebella were reduced in size and lacked surface foliation (Fig. 4). Upon visual inspection of the samples (n = 3 per group), in contrast to the relative lack of granule neurons, relatively normal numbers of Purkinje cells and neurons of the deep cerebellar nuclei were present in *Atoh1^trhl/trhl^* mice. However, the Purkinje cells, which normally localize beneath the EGL, did not form a distinct, organized layer as seen in 4E.Some Purkinje cells localized improperly at the periphery of the cerebellum while a significant subpopulation even failed to migrate from their initial central area as is evident in the representative section in Figure 4F. Staining with glial fibrillary acidic protein[20] and nestin[21], both of which label glial cells, demonstrated properly localized radial glial cells and no excessive gliosis throughout the cerebella (data not shown). These results suggest that *Atoh1*is essential for formation of the EGL, likely through regulation of downstream effectors. The disorganization of the Purkinje cells in particular may be a function of either the *Atoh1* gene's effect on cell migration or due to the direct disturbance of the EGL. In *Atoh1^trhl/trhl^* mice, absence of the EGL led to a lack of foliation of the cerebella, which was typically observable in stained sections from normal embryos (n = 3 per group) starting from day 18 of the embryonic period (E18). The observations in this mutant mouse are thus consistent with those of Ben-Arie and colleagues in the *Atoh1*-null mutant mouse with concern to the cerebellum [2] and the model can be further used to investigate postnatal function and development because of vitality beyond birth.

### Progressive hearing loss indicated by ABR and DPOAE

**Figure 5:**
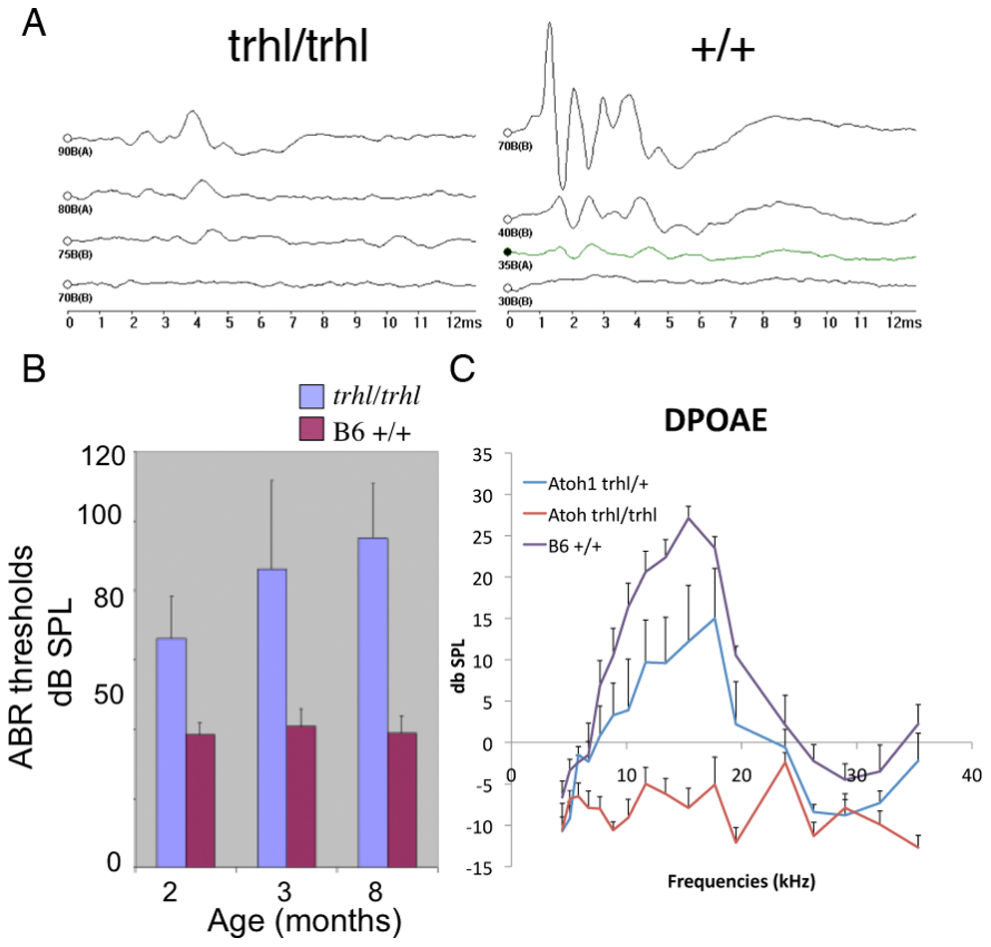
Hearing tests indicate progressive hearing loss in mutants. (**A**)Representative ABR thresholds (with click) from wild-type B6 (right panel) and mutant *Atoh1^trhl/trhl^*(left panel) mice recorded at 21 days of age (P21); n = 5 were tested for each genotype. Note the differences in threshold, waveform morphology and amplitude of the peaks. The X-axis represents time in milliseconds and Y-axis represents amplitude of the action potentials in microvolts. Each waveform, at a different, declining dB level to examine response, is stacked onto the same graph. The right panel displays a much stronger response to the stimulus and clear waveforms at 70 dB but the left panel does not show a very strong response even at 90 dB. (**B**)Average of ABR thresholds evoked by 16 kHz pure tone recorded from wild-type and *Atoh1^trhl/trhl^*mice at ages 2, 3, and 8 months (n = 5 for each grouping). ABR threshold was higher for mutant strains and progressively worsened over the measured time period, in comparison to littermates. Error bars represent standard deviation. The graph in (**C**) showsDPOAE amplitudes in mice of three different genotypes at P80 (n = 10 for each genotype). DPOAE amplitudes of the homozygous mutant mice were undetectable at all frequencies while B6 +/+ mice had normal DPOAE amplitudes. The heterozygous had minor reduction of DPOAE amplitudes at 15-20 kHz. Error bars indicate standard error at each time point, for each mouse group.

To test hearing and to detect any progressive changes, we measured auditory brainstem response (ABR) thresholds over time, beginning at 21 days postnatum (P21) and at a number of time points up to 420 days. Measurement of hearing at P21 indicated a higher threshold and lower amplitude with more prominent central peaks in the *Atoh1^trhl/trhl^* strain, in comparison to normal and heterozygous mice (n = 5 per group; Fig. 5A). It is important to note that the background strain of mice C57BL/6J has an underlying hearing loss mutation of *Cdh23^753A^*that causes progressive hearing loss and deafness starting at 10 months of age. Our data isconsistent with hair cell loss and possible auditory neuron loss in *Atoh1^trhl/trhl^* mice that is starting much earlier as evidenced by issues with hearing at the first P21 time point and most likely related to the induced hypomorphic Atoh1 mutation.We found the sensorineural hearing loss to be slowly progressive, with a phase of accelerated loss at approximately 3 months postnatal (Fig. 5B). The sensorineural hearing loss and its progression were more prominent at higher frequencies, corresponding to hair cell loss in the basal cochlear region.Distortion product otoacoustic emission (DPOAE) recordings (Fig. 5C) showed a comparative decrease in the homozygous Atoh1 vs. heterozygous Atoh1 mutant vs. the B6 +/+ control (n = 5 per group). The reduced DPOAE is indicative of the expected reduced function of the inner ear and its hair cells. This decline corresponded to the histological observations about the loss of hair cells, more in the Atoh1 homozygous mutants than heterozygous (Fig. 2) as well as ABR recordings.

### Biological Significance, Clinical Significance, and Future Prospects

Since this model displays hair cell loss and neuronal alteration, it is important to understand the molecular mechanisms that underlie specification and differentiation of immature and/or proliferating epithelial cells into mature hair cellstogether withcomponents of relevant neuronal pathways. This knowledge may allow elucidation of the requirements for triggering cell repair and/or regeneration after injury. Identification and manipulation of candidate genes that control and regulate hair cell differentiation would facilitate restoration of hair cells in a damaged organ of Corti. The homozygous *Atoh1^trhl^*mouse thus provides a valuable model to study the effects of the *Atoh1* gene on postnatal ear anatomy and function. It also provides a good model for studying gene therapy, as*Atoh1* gene transfer could be a potential treatment to promote hair cell regeneration[8], [9], [22]. The role of *Atoh1* was explored byBerminghamand colleagues[7] who demonstrated that at embryonic day 18.5, *Atoh1* expression is restricted to the hair cells of the developing sensory epithelia. However, using quantitative real time-polymerase chain reaction (RT-PCR) analysis, Zheng[23]showed that *Atoh1* is expressed in adults, albeit at lower levelsthan during development. The progressive hearing dysfunction and loss of hair cellsin*trhl/trhl* mice suggests that *Atoh1* is required not only for formation but also for maintenance of hair cells in adult mice. The mutated *Atoh1* gene might cause long-term or permanent expression changes of downstream genes such as LIM homeobox protein 2 *(Lh2a)*, LIM homeobox protein 9 *(Lhx9)*, BarH-like 1 *(Barhl1)*and BarH-like 2 *(Barhl2)* [24], [25], which can be investigated in future studies. *Barhl1*has been extensively studied and appears to play an essential role in the migration and survival of cerebellar and pre-cerebellar neurons in mice as well as a rolein normal hair cell function where it is expressed in inner ear hair cells, but most abundantly in cochlear outer hair cells[20], [21], [26], [27].Progressive loss of cochlear hair cells in *Barhl1*-null mice indicates a crucial role for *Barhl1*in the maintenance of these sensory cells. The onset of hair cell defects in the *Barhl1* ^–/–^resulting in early low frequency loss and progressive loss around 3 months [20]suggests that *Barhl1* is likely to act downstream of *Atoh1*-dependentinner ear development which is sufficient for the initial generation of those hair cells even in the absence of *Barhl1*expression. Similarly, analysis of *Barhl2*-flanking sequences identified an enhancer that can be activated by the basic helix-loop-helix factor Atoh1 and can drive spinal cord-specific gene expression [25].*Atoh1* must be expressed in sufficient quantity to appropriately act on downstream genes such as *Barhl1* and *Barhl2*, but *Atoh1* over-expression has been associated with increased granule neuron precursor proliferation and also with medulloblastoma, one of the most common pediatric nervous system tumors [11]. Additional research is needed to explore these associations to reach a better understanding of the interplay between these factors to better direct possible routes of future therapies. Based on a exclusive search in Pubmed, OMIN (http://www.ncbi.nlm.nih.gov/omim?term=atoh1), http://www.hgvs.org/dblist/dblist.html and genecards.org search, there is no survival human mutation although many polymorphism in human genome (including 32 SNPs- Single Nucleotide Polymorphismand one SSLP-Single Sequence Length Polymorphisms) in ATOH1.

According to informatics.jax.org (http://www.informatics.jax.org/marker/MGI:104654),all 9 alleles of *Ahoh1* aretargeted alleles, and they either die early in life or need complicated breeding/gene manipulation to order show desired phenotype. This makes the *Atoh1^trhl^*mousewith normal lifespan an uniquely valuable model to study the effects of the *Atoh1* gene on postnatal ear anatomy and function in hearing, central nerves system and cancer biology.

## Conclusion

Since the *trhl* mutant mice have a normal life span, unlike previous *Atoh1*-nullmutations, which are perinatally lethal, we can take advantage of this newmodel to investigate many aspects of downstream hearing and balance pathophysiology, molecular biology, proteomics and cancer biology. We feel that this unique model can offer an interesting alternate pathway to examine *Atoh1* function to complement other models and not necessarily replace them. It is important to note that this model is a hypomorphic mutation and that *Atoh1* has more of an effect developmentally than on the neurogenesis and inner ear hair cell formation [1], [3]. The effect of this mutation on other *Atoh1*affected tissues is currently unknown but is an important avenue to investigate in the future. Inaddition to the normal life span, the *Atoh1*mutant mice display unique features as a model for progressive age related hearing loss. These characteristics may reflect a crucial requirement for *Atoh1*in the lifelong maintenance of cochlear hair cells. Conceivably, an understanding of this requirement at the molecular level will provide significant insights into the pathology of aging-related deafness, creating further demand for this model as a significant tool to explore human diseases including lung cancers[19], intestinal tumorigenesis, [5] and neuroblastomaformation [19], [28].
